# Allergic diseases of the skin and drug allergies – 2016. Ranitidine treatment in preterm infants and the prevalence of atopy at two years of age

**DOI:** 10.1186/1939-4551-6-S1-P103

**Published:** 2013-04-23

**Authors:** Than Soe, Lucy Bemand-Qureshi, Sadaf Chaudhry, Vijay Chakravarti

**Affiliations:** 1 Child Health, Princess Alexandra Hospital, Harlow, UK

## Background

Acid suppression treatment has been linked to an increased incidence of allergy in adults. We evaluate how ranitidine, widely used off-label to treat gastro-esophageal reflux symptoms in neonates, may impair peptic digestion, increasing the risk of sensitization to digestion-labile food antigens and thus increasing the prevalence of atopy.

## Methods

We carried out a retrospective review of preterm infants of between 26 and 37 weeks gestation admitted over a two-year period between April 2008 and March 2010. Those preterm infants treated with the H2-receptor antagonist ranitidine for more than seven days were identified. A control group of preterm infants who did not receive treatment was selected by matching gestation, birth weight and disease severity using a validated scoring method. We analysed both maternal data (mode of delivery, use of intrapartum antibiotics, prolonged rupture of the membranes, sepsis) and neonatal data (birth weight, gestation, interventions, sepsis, type of feeding and neonatal complications). Information concerning the development of atopy in children in both groups, as well as any family history of atopy, was obtained through a simple questionnaire.

## Results

There were 38 infants in the group treated with ranitidine (exposure group) and 37 in the control group**.** There was no significant difference in perinatal characteristics and neonatal morbidity between the two groups**.** The prevalence of atopy was 39% in the exposure group and 47% in the control group (*p*= 0.57). Subgroup analysis showed the prevalence of milk allergy was 17% in both groups and *N*on *S*ignificant (NS); the prevalence of atopic eczema was 18% (exposure) vs 45% (control) (*p* = <.001); and the prevalence of recurrent wheeze or asthma was 13% (exposure) vs 11% (control) (*NS*). Prevalence of other food allergies was very low and comparable. No difference in the family history of atopy was observed.

## Conclusions

Ranitidine treatment in preterm infants did not increase the overall prevalence of atopy at two years of age but a significantly lower prevalence of atopic dermatitis was observed in the group treated with ranitidine.

